# Supplementary material for: Igh and Igk loci use different folding principles for V gene recombination due to distinct chromosomal architectures of pro-B and pre-B cells
Source: Nat Commun. 2023 Apr 21;14:2316. doi: 10.1038/s41467-023-37994-9 (PMC10121685; doi:10.1038/s41467-023-37994-9)
Supplement: Supplementary file 1 — Supplementary Information file [file 41467_2023_37994_MOESM1_ESM.pdf]

SUPPLEMENTARY INFORMATION

Igh and *Igk* loci use different folding principles for V gene recombination due to distinct chromosomal architectures of pro-B and pre-B cells

Louisa Hill[1], Gordana Wutz[1], Markus Jaritz[1], Hiromi Tagoh[1], Lesly Calderón[1], Jan-Michael Peters[1], Anton Goloborodko[2], and Meinrad Busslinger[1]

[1] Research Institute of Molecular Pathology (IMP), Vienna BioCenter (VBC), Campus-Vienna-Biocenter 1, A-1030 Vienna, Austria

[2] Institute of Molecular Biotechnology (IMBA), Austrian Academy of Sciences, Vienna BioCenter (VBC), Dr. Bohr-Gasse 3, A-1030 Vienna, Austria

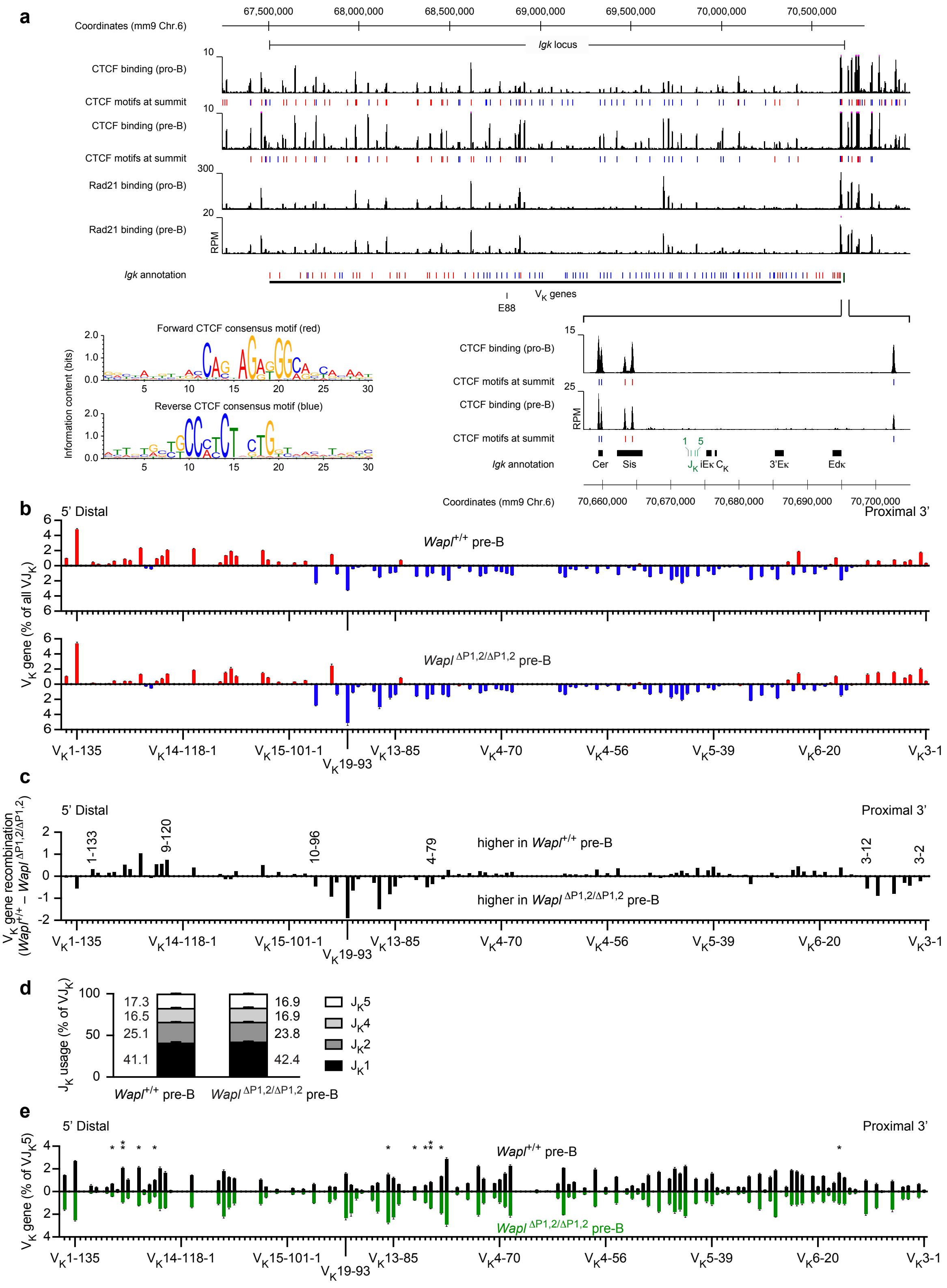

Supplementary Figure 1

Supplementary Fig. 1 | CTCF binding and V_K-J_K recombination at the *Igk* locus in pre-B cells. **a**, Orientation of the CTCF-binding sites and V_K genes in the *Igk* locus. The binding patterns of CTCF and cohesin (Rad21) at the *Igk* locus were determined by ChIP-seq analysis of short-term IL-7-cultured *Rag2*$^{-/-}$ pro-B cells (Medvedovic et al., Immunity 39, 229-244) and *ex vivo* sorted *Rag2*$^{-/-}$ pre-B cells expressing a rearranged Igμ transgene (Loguercio et al., Front. Immunol. 9, 425). Along the V_K gene cluster, a total sum of 67 CTCF-binding sites (CBEs) were identified in pro-B and pre-B cells (see Methods). The forward and reverse consensus CTCF-binding motifs shown were determined by analyzing the CBEs at the *Igk* locus with the *de novo* motif discovery program MEME. The locations of forward (red) and reverse (blue) CTCF-binding motifs, which were detected at the summit of the CTCF peaks, are shown together with the locations of forward (red)- and reverse (blue)-oriented V_K genes and the E88 enhancer, based on the annotation of the C57BL/6 *Igk* locus (mm9 Chr. 6; 67,505,630 [V_K2-137] – 70,694,944 [Edκ]) (Supplementary Data S1a). The J_K and C_K elements and the regulatory elements Cer, Sis, iEκ, 3'Eκ and Edκ in the 3' proximal *Igk* domain are shown at higher magnification together with the respective CTCF-binding pattern. It is important to note that pro-B cells, which are short-term cultured with IL-7, resemble pre-B cells rather than pro-B cells with regard to their CTCF-binding pattern and epigenetic landscape at the *Igk* locus (Loguercio et al., Front. Immunol. 9, 425). **b**, V_K gene recombination in *ex vivo* sorted *Wapl*$^{+/+}$ and *Wapl*$^{\Delta P1,2/\Delta P1,2}$ pre-B cells, as determined by VDJ-seq analysis. The data of Fig. 1b were replotted to indicate the frequency of the forward (red)- and reverse (blue)-oriented V_K genes above and below the line, respectively. The recombination frequency of each V_K gene is indicated as percentage of all V_K-J_K rearrangement events and is shown as mean value with SEM, based on 4 independent VDJ-seq experiments for each pre-B cell type. The different V_K genes (horizontal axis) are aligned according to their position in the *Igk* locus (Supplementary Data 1a). **c**, Difference of V_K gene usage across the *Igk* locus, which was determined for each V_K gene by subtracting its mean recombination frequency in *Wapl*$^{\Delta P1,2/\Delta P1,2}$ pre-B cells from that in *Wapl*$^{+/+}$ pre-B cells. The 3' and 5' ends of a region containing differentially recombined V_K genes are indicated by their respective V_K genes. **d**, The usage of the 4 functional J_K elements is indicated as relative frequency of all V_K-J_K rearrangements detected in *Wapl*$^{+/+}$ and *Wapl*$^{\Delta P1,2/\Delta P1,2}$ pre-B cells, respectively, and is shown as mean value with SEM, based on 4 independent VDJ-seq experiments for each pre-B cell type. **e**, Analysis of the V_K gene rearrangements involving the most 3' proximal J_K5 element in *ex vivo* sorted *Wapl*$^{+/+}$ (black) and *Wapl*$^{\Delta P1,2/\Delta P1,2}$ (green) pre-B cells. The recombination frequency of each V_K gene is indicated as percentage of all V_K-J_K5 rearrangements determined for each pre-B cell type and is shown as mean value with SEM, based on 4 independent VDJ-seq experiments for each pre-B cell type. Statistical data were analyzed by multiple *t*-tests (unpaired and two-tailed) with Holm-Šídák correction; $*P < 0.05$, $**P < 0.01$. Source data are provided in the Source Data file.

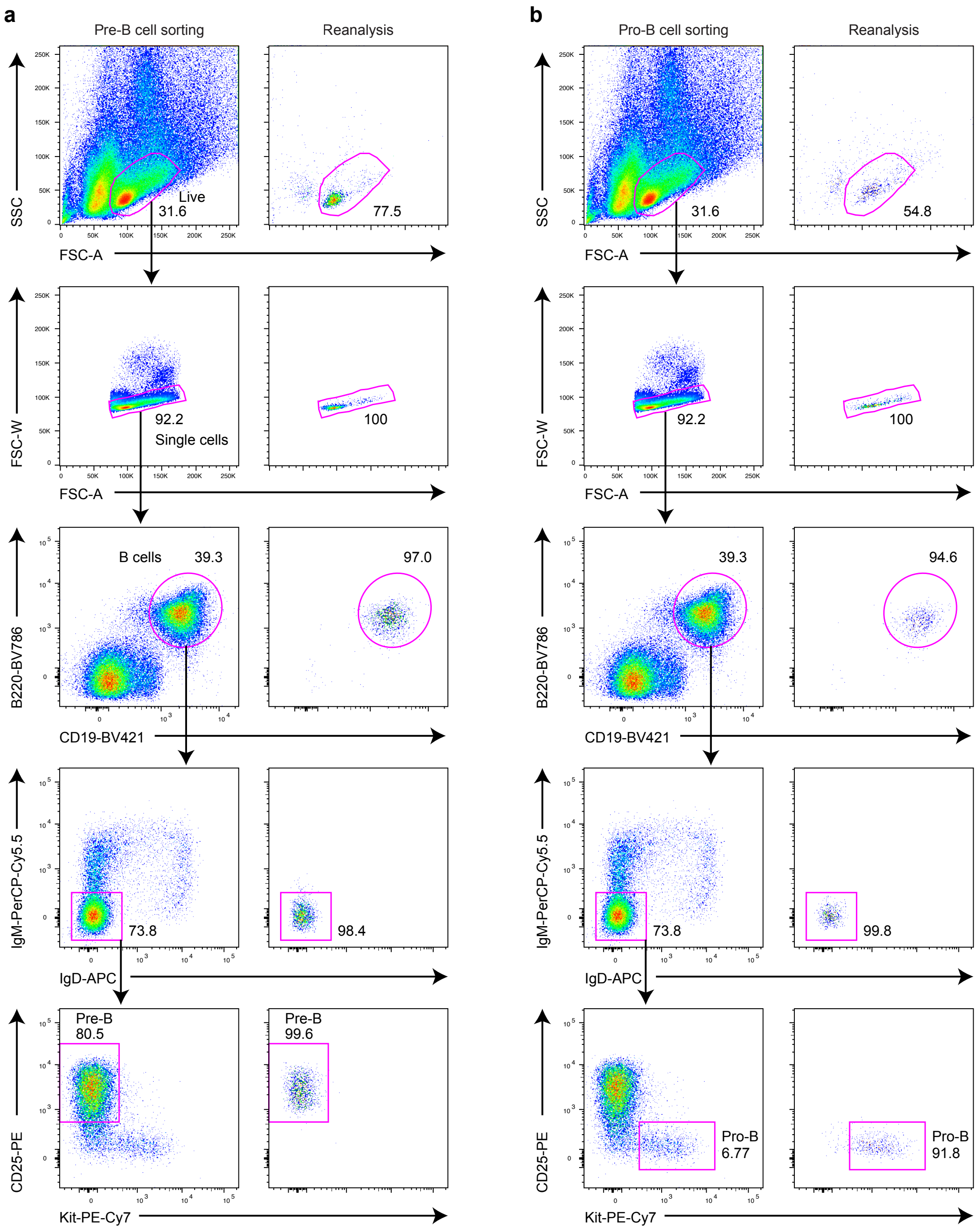

Supplementary Fig. 2 | Flow-cytometric sorting of pro-B and pre-B cells.

a, Pre-B cells were isolated by flow-cytometric sorting as $CD19^{+}B220^{+}IgM^{-}IgD^{-}Kit^{-}CD25^{+}$ cells from the bone marrow of *Wapl*$^{+/+}$, *Wapl*$^{\Delta P1,2/\Delta P1,2}$ and *Igh*$^{B1\text{-}8hi/+}$ *Rag2*$^{-/-}$ mice at the age of 4-6 weeks. **b**, Pro-B cells were isolated as $CD19^{+}B220^{+}IgM^{-}IgD^{-}Kit^{+}CD25^{-}$ cells from the bone marrow of *Wapl*$^{+/+}$, *Wapl*$^{\Delta P1,2/\Delta P1,2}$ and *Rag2*$^{-/-}$ mice at the age of 4-6 weeks. The different gates used for flow-cytometric sorting are indicated. The purity of the sorted cell populations was determined by flow-cytometric reanalysis. The percentage of cells in the different gates is shown.

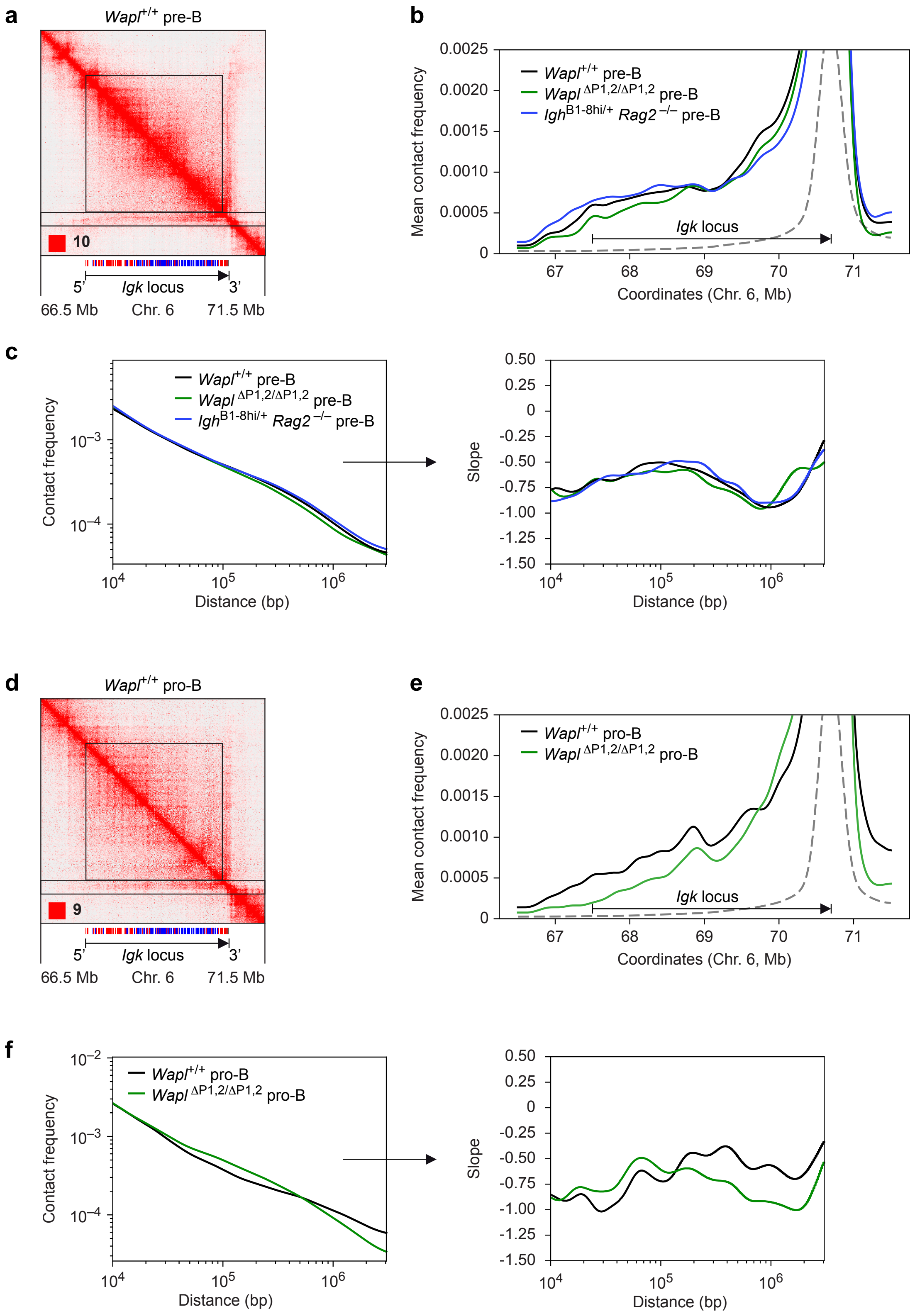

Supplementary Figure 3

Supplementary Fig. 3 | Quantification of the Hi-C interaction frequencies at the *Igk* locus in pro-B and pre-B cells. **a**, The Hi-C contact matrix of *Wapl*$^{+/+}$ pre-B cells (Fig. 1d) is shown as an example to indicate, which area of the Hi-C interaction stripe (emanating from the *Igk* 3' end) was analyzed by contact frequency measurements in (**b**) and which area (square) within the *Igk* locus was used for calculating the contact frequency plots shown in (**c**). **b**, Mean contact frequencies along the Hi-C interaction stripe at the *Igk* locus in *Wapl*$^{+/+}$ (black), *Wapl*$^{\Delta P1,2/\Delta P1,2}$ (green) and *Igh*$^{B1\text{-}8hi/+}$ *Rag2*$^{-/-}$ (blue) pre-B cells (corresponding to the data shown in Figure 1d). The dotted line indicates the mean contact frequencies of 100 controls, starting at random positions from the diagonal of the Hi-C contact matrix of chromosome 6 in the same direction as the *Igk* stripe. The mm9 coordinates of chromosome 6 are shown. The 5' – 3' orientation of the *Igk* locus is indicated by an arrow. **c**, Frequency distribution of contacts within the *Igk* locus as a function of the genomic distance. The contact frequency plot (left) is shown for *Wapl*$^{+/+}$ (black), *Wapl*$^{\Delta P1,2/\Delta P1,2}$ (green) and *Igh*$^{B1\text{-}8hi/+}$ *Rag2*$^{-/-}$ (blue) pre-B cells, while the first derivatives (slope) of the contact frequency curves are indicated to the right (see Methods). **d**, The Hi-C contact matrix of *Wapl*$^{+/+}$ pro-B cells (Fig. 4b) is shown with the area of the Hi-C interaction stripe and area (square) within the *Igk* locus that were used for calculating the contact frequency plots shown in (**e**) and (**f**), respectively. **e**, Mean contact frequencies along the Hi-C interaction stripe in *Wapl*$^{+/+}$ (black) and *Wapl*$^{\Delta P1,2/\Delta P1,2}$ (green) pro-B cells (corresponding to the data shown in Figure 4b). The dotted line indicates the mean contact frequencies of 100 controls that were obtained as described in (**b**). **f**, Frequency distribution of contacts within the *Igk* locus as a function of the genomic distance. The contact frequency plot (left) is shown for *Wapl*$^{+/+}$ (black) and *Wapl*$^{\Delta P1,2/\Delta P1,2}$ (green) pro-B cells, while the first derivatives (slope) of the contact frequency curves are indicated to the right. All curves have been smoothened.

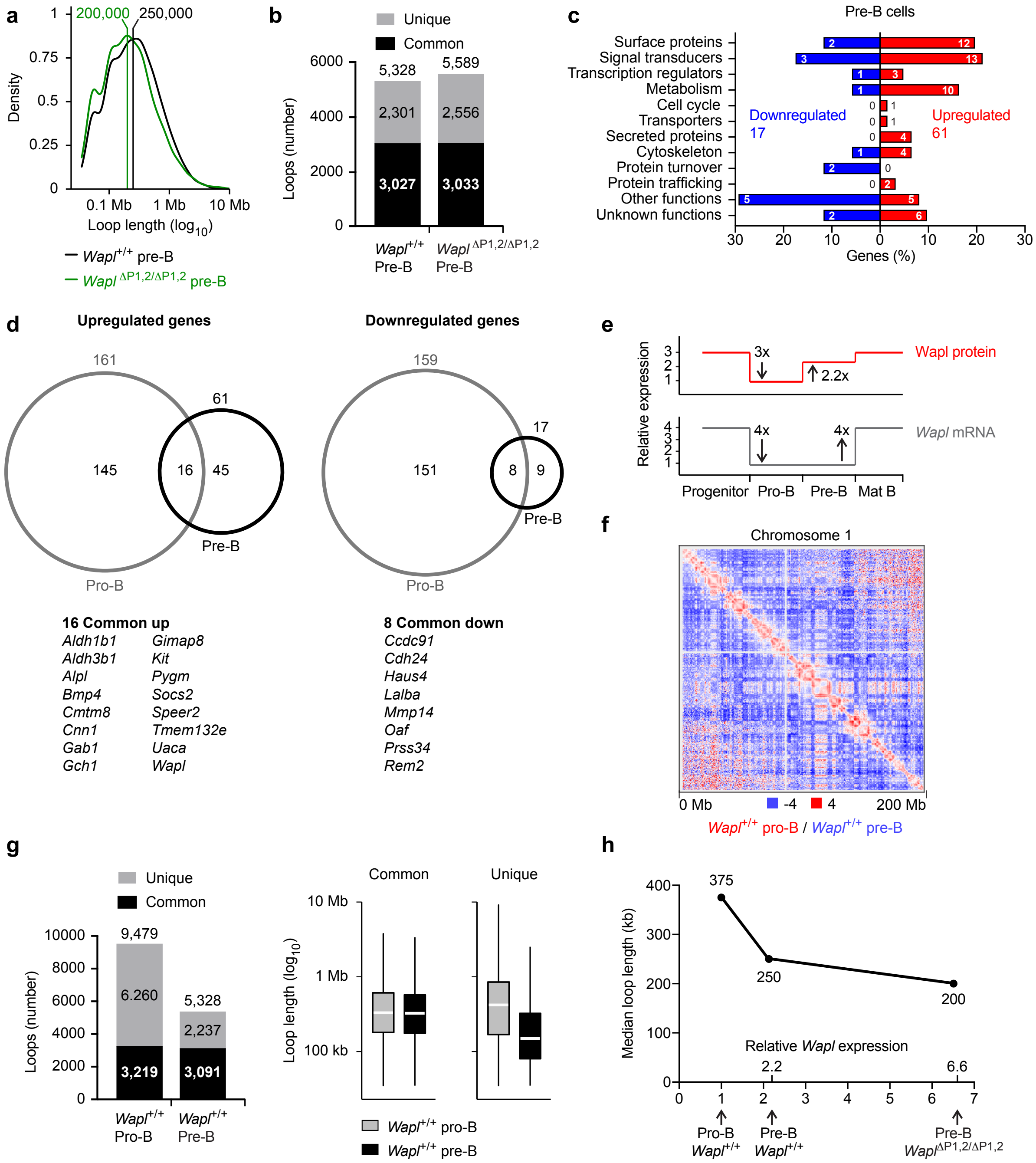

Supplementary Figure 4

Supplementary Fig. 4 | Chromosomal architecture and gene expression in *Wapl*$^{+/+}$ and *Wapl*$^{\Delta P1,2/\Delta P1,2}$ pre-B cells. **a**, Density distribution of the loop length in *Wapl*$^{+/+}$ (black) and *Wapl*$^{\Delta P1,2/\Delta P1,2}$ (green) pre-B cells, as determined with HiCCUPS of Juicer (see Methods). The median loop length (in kb) is shown for each genotype. **b**, Number of common and unique loops determined by Hi-C analysis of *Wapl*$^{+/+}$ and *Wapl*$^{\Delta P1,2/\Delta P1,2}$ pre-B cells (see Methods). **c**, Functional classification and quantification of the proteins that are encoded by up-regulated (red) and down-regulated (blue) genes in *Wapl*$^{\Delta P1,2/\Delta P1,2}$ pre-B cells relative to *Wapl*$^{+/+}$ pre-B cells (Fig. 2f, Supplementary Data 2). The bar size indicates the percentage of up- or down-regulated genes in each functional class relative to the total up- or down-regulated genes, respectively. Numbers in the bars indicate the number of genes in each functional class. **d**, Venn diagrams indicating the overlap between genes that were > 2-fold up- or down-regulated in *Wapl*$^{\Delta P1,2/\Delta P1,2}$ pro-B cells relative to *Wapl*$^{+/+}$ pro-B cells or in *Wapl*$^{\Delta P1,2/\Delta P1,2}$ pre-B cells relative to *Wapl*$^{+/+}$ pre-B cells. The RNA-seq analyses of all 4 cell types were performed at the same time and have already been published (Hill et al., Nature 584, 142-147). The number of differentially regulated genes identified in pro-B or pre-B cells is shown above each circle. Genes that were up- or down-regulated in both pro-B and pre-B cells are indicated below. **e**, Schematic diagram indicating the relative expression levels of the Wapl protein and *Wapl* mRNA, based on the data shown in Fig. 3a and previously published data (Hill et al., Nature 584, 142-147). **f**, Differential Hi-C contact matrix of chromosome 1 displaying the difference in pixel intensity between *Wapl*$^{+/+}$ pro-B and *Wapl*$^{+/+}$ pre-B cells as the ratio (pro-B) / (pre-B). More interactions (red) in the TAD range were observed for *Wapl*$^{+/+}$ pro-B cells, while more interactions (blue) in the compartment range were detected for *Wapl*$^{+/+}$ pre-B cells. **g**, Number (left) and length (right) of common and unique loops determined by Hi-C analysis of *Wapl*$^{+/+}$ pro-B and *Wapl*$^{+/+}$ pre-B cells (see Methods). The white lines (right) indicate the median value, and boxes represent the middle 50% of the data. Whiskers denote all values of the 1.5× interquartile range. The median loop length is 330 kb (common) and 420 kb (unique) in *Wapl*$^{+/+}$ pro-B cells (gray) and 325 kb (common) and 150 kb (unique) in *Wapl*$^{+/+}$ pre-B cells (black). **h**, Exquisite Wapl-dosage sensitivity of genome-wide chromatin looping during the transition from pro-B to pre-B cells. The median loop length determined in *Wapl*$^{+/+}$ pro-B, *Wapl*$^{+/+}$ pre-B and *Wapl*$^{\Delta P1,2/\Delta P1,2}$ pre-B cells (Fig. 2e and Fig. 3e) is plotted against the relative Wapl protein expression in these three cell types (Fig. 1a and Fig. 3a), with the Wapl expression level in *Wapl*$^{+/+}$ pro-B cells being set to 1. Source data are provided in the Source Data file.

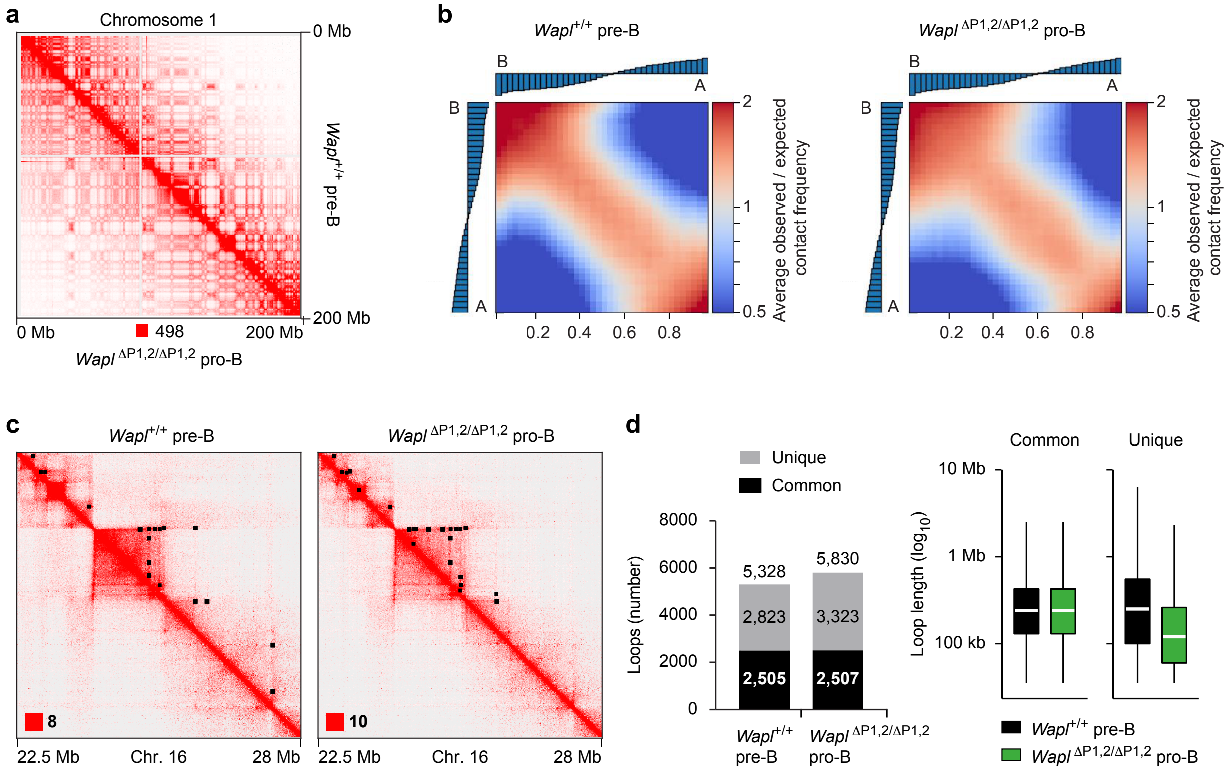

Supplementary Fig. 5 | Similar chromosomal architectures of *Wapl*$^{\Delta P1,2/\Delta P1,2}$ pro-B and *Wapl*$^{+/+}$ pre-B cells. **a**, Hi-C contact matrices of chromosome 1, determined for short-term cultured *Wapl*$^{\Delta P1,2/\Delta P1,2}$ pro-B cells (Hill et al., Nature 584, 142-147) and *ex vivo* sorted *Wapl*$^{+/+}$ pre-B cells (this study) are plotted at a 500-kb bin resolution with Juicebox. The intensity of each pixel represents the normalized number of contacts between a pair of loci. The maximum pixel intensity is indicated below (red square). **b**, Compartmentalization saddle plots indicating the average intrachromosomal interaction frequencies between loci (40-kb bins) that are normalized by the expected interaction frequency based on genomic distance. Bins are sorted by their PC1 (eigenvector) value derived from Hi-C data that were obtained with *Wapl*$^{\Delta P1,2/\Delta P1,2}$ pro-B or *Wapl*$^{+/+}$ pre-B cells (see Methods). Preferential interactions (B-B) between B-type compartments are in the upper left corner and A-A interactions in the lower right corner. **c**, Hi-C contact matrices of a zoomed-in region on chromosome 16 (mm9; 22,500,000 – 28,000,000), shown for *Wapl*$^{+/+}$ pre-B and *Wapl*$^{\Delta P1,2/\Delta P1,2}$ pro-B cells. Black dots indicate loop anchors identified with HiCCUPS of Juicebox. **d**, Number (left) and length (right) of common and unique loops determined by Hi-C analysis of *Wapl*$^{+/+}$ pre-B and *Wapl*$^{\Delta P1,2/\Delta P1,2}$ pro-B cells (see Methods). The white lines (right) indicate the median value, and boxes represent the middle 50% of the data. Whiskers denote all values of the 1.5× interquartile range. The median loop length is 240 kb (common) and 250 kb (unique) in *Wapl*$^{+/+}$ pre-B cells (black) and 240 kb (common) and 120 kb (unique) in *Wapl*$^{\Delta P1,2/\Delta P1,2}$ pro-B cells (green). Source data are provided in the Source Data file.

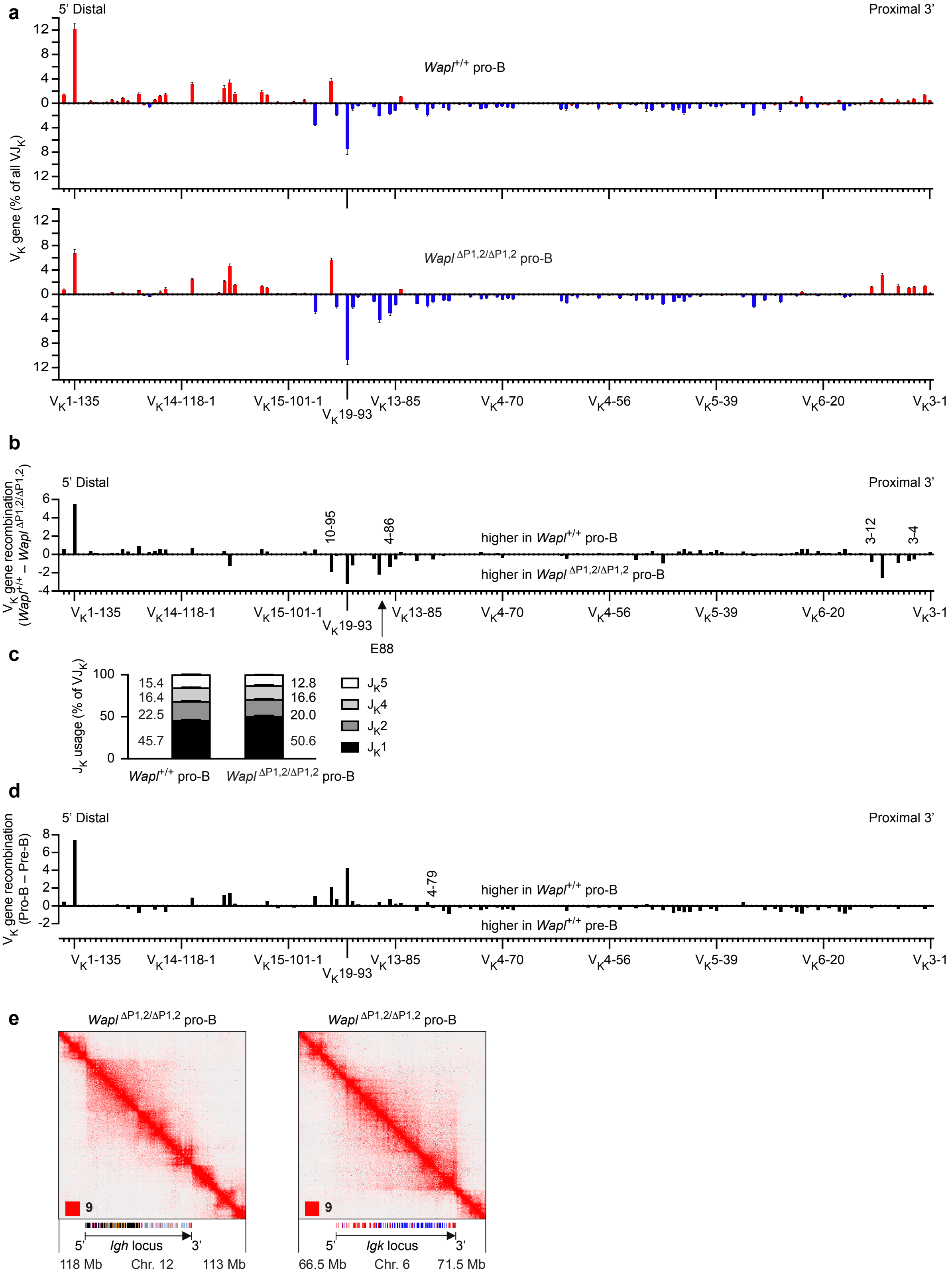

Supplementary Figure 6

Supplementary Fig. 6 | V_K-J_K rearrangements in pro-B cells and long-range interactions at *Igh* and *Igk* loci in *Wapl*$^{\Delta P1,2/\Delta P1,2}$ pro-B cells. **a**, V_K gene recombination analysis of *ex vivo* sorted *Wapl*$^{+/+}$ and *Wapl*$^{\Delta P1,2/\Delta P1,2}$ pro-B cells. The VDJ-seq data of Fig. 4a were replotted to indicate the frequency of the forward (red)- and reverse (blue)-oriented V_K genes above and below the line, respectively. The recombination frequency of each V_K gene is indicated as percentage of all V_K-J_K rearrangement events and is shown as mean value with SEM, based on 6 independent VDJ-seq experiment for each pro-B cell type. The different V_K genes (horizontal axis) are aligned according to their position in the *Igk* locus (Supplementary Data 1a). **b**, Difference of V_K gene usage across the *Igk* locus, which was determined for each V_K gene by subtracting its mean recombination frequency in *Wapl*$^{\Delta P1,2/\Delta P1,2}$ pro-B cells from that in *Wapl*$^{+/+}$ pro-B cells. The 3' and 5' ends of a region containing differentially recombined V_K genes are indicated by their respective V_K genes. **c**, The usage of the 4 functional J_K elements is indicated as relative frequency of all V_K-J_K rearrangements detected in *Wapl*$^{+/+}$ and *Wapl*$^{\Delta P1,2/\Delta P1,2}$ pro-B cells, respectively, and is shown as mean value with SEM, based on 6 independent VDJ-seq experiments for each pro-B cell type. **d**, Difference of V_K gene usage in the V_K gene cluster, which was determined for each V_K gene by subtracting its mean recombination frequency in *Wapl*$^{+/+}$ pre-B cells from that in *Wapl*$^{+/+}$ pro-B cells. **e**, Comparison of the Hi-C contact matrices at the *Igh* and *Igk* loci in *Wapl*$^{\Delta P1,2/\Delta P1,2}$ pro-B cells, based on published Hi-C data (Hill et al., Nature 584, 142-147). The orientation and annotation of the *Igk* and *Igh* loci are shown. The maximum pixel intensity is indicated in the lower left of each panel. The genomic coordinates (mm9) of the *Igh* and *Igk* regions are shown. Source data are provided in the Source Data file.

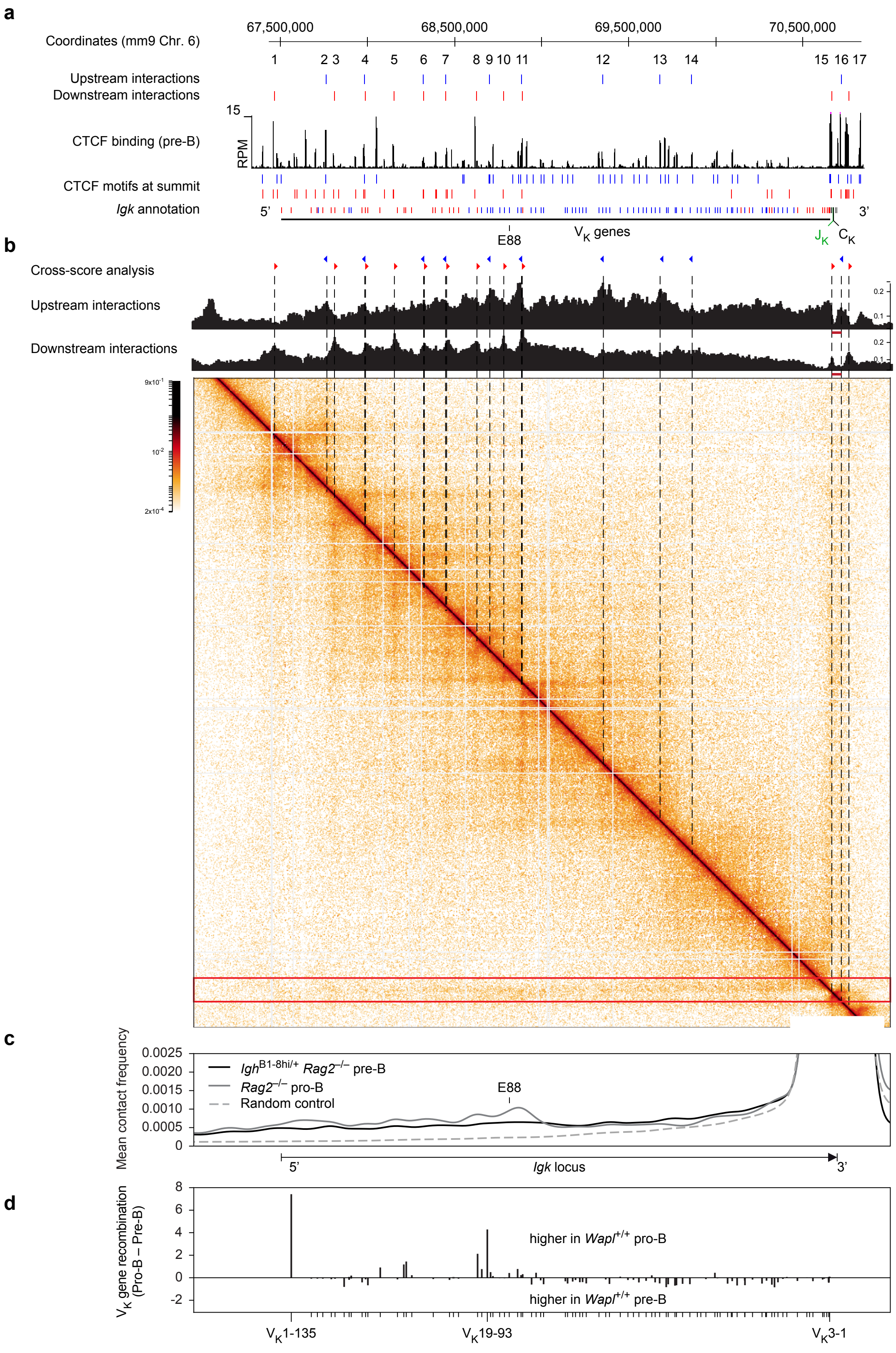

Supplementary Figure 7

Supplementary Fig. 7 | Identification of loop anchors at the *Igk* locus in *Igh*$^{\mathbf{B1\text{-}8hi/+}}$ *Rag2*$^{-/-}$ pre-B cells. **a**, The CTCF-binding pattern of pre-B cells is shown together with the location of the upstream (blue) and downstream (red) interactions, defined in **b**, and the annotation of the reverse (blue)- and forward (red)-oriented CBEs and V_K genes. The mm9 coordinates of chromosome 6 are indicated. **b**, Identification of upstream and downstream interactions at the base of chromatin loops in the *Igk* locus, as determined by Micro-C analysis of *Igh*$^{B1\text{-}8hi/+}$ *Rag2*$^{-/-}$ pre-B cells (Fig. 5b, shown below at higher magnification) and subsequent analysis with the Cross-score program (see Methods). Peak calling of the profile defined by the Cross-score interaction values (black) identified at least 17 peaks that corresponded to upstream or downstream interactions at loop anchors and colocalized with the observed internal stripes of the Micro-C pattern at the *Igk* locus. The upstream and downstream interactions are denoted by blue and red arrowheads, respectively. The position of the relatively stable 'regulatory' loop containing the J_K, C_K and *Igk* enhancer elements is indicated by a red horizontal bar below the interaction plot. The area of the stripe, which was analyzed by contact frequency measurements in **c**, is indicated by a red box. **c**, Mean contact frequencies along the stripe originating from the 3' end of the *Igk* locus (red box in **b**) in *Rag2*$^{-/-}$ pro-B cells (gray) and *Igh*$^{B1\text{-}8hi/+}$ *Rag2*$^{-/-}$ (black) pre-B cells (corresponding to the Micro-C data shown in Figure 5a,b). The dotted line indicates the mean contact frequency of 100 random controls that were generated as described in Supplementary Fig. 3b. The 5' – 3' orientation of the *Igk* locus is indicated by an arrow, and the position of the E88 enhancer is shown. **d**, Differential V_K gene usage in *Wapl*$^{+/+}$ pro-B cells and *Wapl*$^{+/+}$ pre-B cells, as determined by subtraction of the mean recombination frequency of each V_K gene between the two cell types (described in Supplementary Fig. 6d). The V_K gene usage is plotted at the position of the corresponding V_K gene in the *Igk* locus.

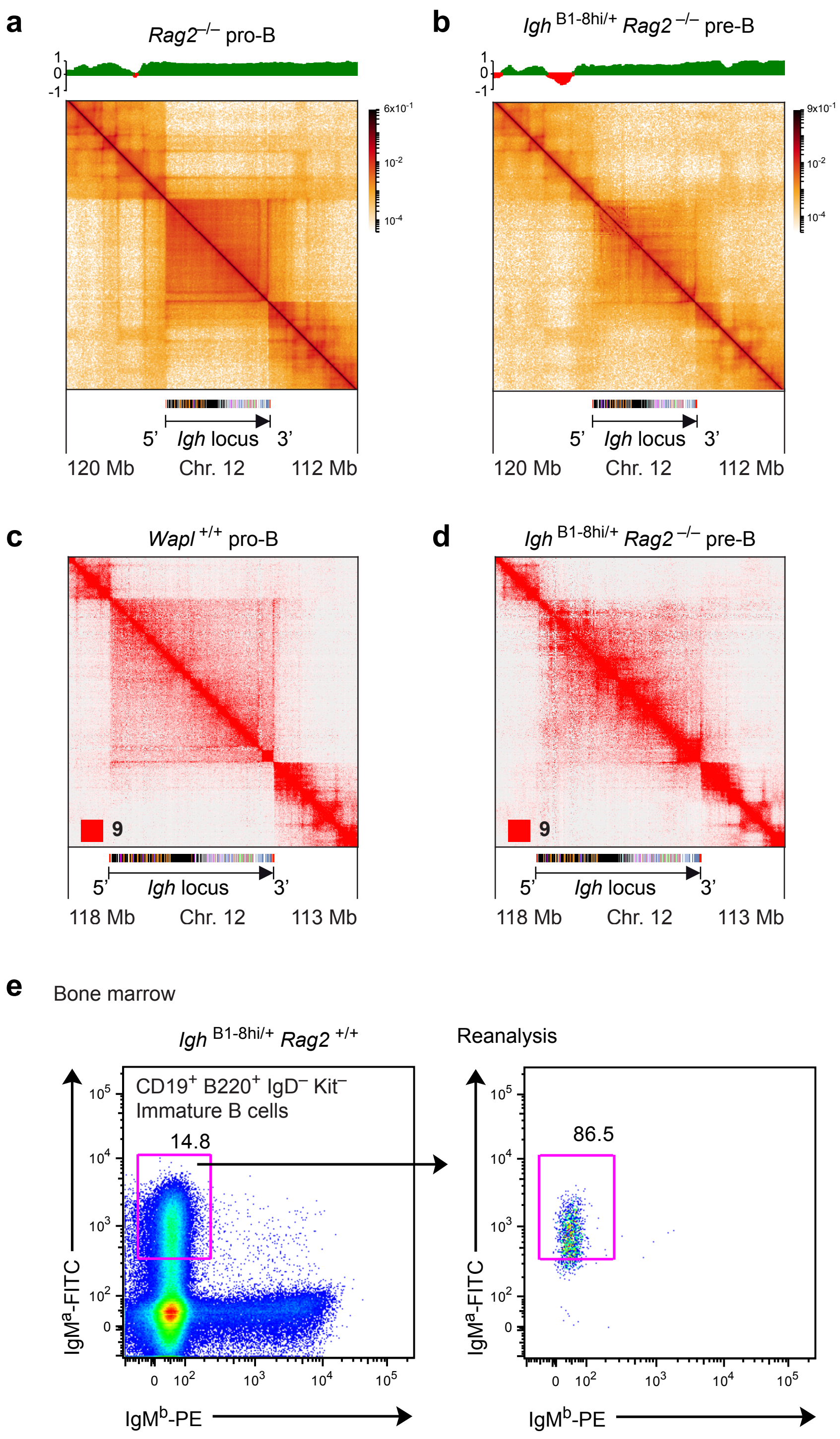

Supplementary Figure 8

Supplementary Fig. 8 | Long-range interactions at the *Igh l*ocus and surrounding regions in pro-B and pre-B cells and allelic exclusion of V_H gene recombination in *Igh*$^{\mathbf{B1\text{-}8hi/+}}$ pre-B cells. **a, b,** Micro-C analysis of *ex vivo* sorted *Rag2*$^{-/-}$ pro-B cells (**a**) and *Igh*$^{B1\text{-}8hi/+}$ *Rag2*$^{-/-}$ pre-B cells (**b**). The contact matrices of a zoomed-out region around the *Igh* region are shown together with a plot of the PC1 interaction scores defining the compartments A (green) and B (red). The annotation and orientation of the *Igh* locus is shown below together with the genomic coordinates (mm9). For further explanation, see Figs. 5 and 6. **c**, **d**, Hi-C contact matrices of wild-type pro-B cells (**c**) and *Igh*$^{B1\text{-}8hi/+}$ *Rag2*$^{-/-}$ pre-B cells (**d**). The intensity of each pixel represents the normalized number of contacts between a pair of loci. The maximum intensity is indicated in the lower left of each panel. **e**, Flow cytometric sorting of immature IgM^{a} B cells (CD19^{+}B220^{+}IgD^{-}Kit^{-}) from the bone marrow of *Igh*$^{B1\text{-}8hi/+}$ *Rag2*$^{+/+}$ mice, followed by reanalysis of the sorted cells. The *Igh*$^{B1\text{-}8hi}$ allele expresses IgM^{a}, as it was generated by gene targeting in 129/Ola embryonic stem cells (Shih et al., Nat. Immunol. 3, 399-406), whereas IgM^{b} is expressed from the C57BL/6 *Igh*$^{+}$ allele. The percentage of cells in the gate is indicated.
