# Supplementary material for: Igh and Igk loci use different folding principles for V gene recombination due to distinct chromosomal architectures of pro-B and pre-B cells
Source: Nat Commun. 2023 Apr 21;14:2316. doi: 10.1038/s41467-023-37994-9 (PMC10121685; doi:10.1038/s41467-023-37994-9)
Supplement: Supplementary file 3 — Reporting Summary [file 41467_2023_37994_MOESM3_ESM.pdf]

nature portfolio

Corresponding author(s): Meinrad Busslinger

Last updated by author(s): Mar 28, 2023

Reporting Summary

Nature Portfolio wishes to improve the reproducibility of the work that we publish. This form provides structure for consistency and transparency in reporting. For further information on Nature Portfolio policies, see our Editorial Policies and the Editorial Policy Checklist.

Statistics

For all statistical analyses, confirm that the following items are present in the figure legend, table legend, main text, or Methods section.

n/a	Confirmed	
☐	☒	The exact sample size (*n*) for each experimental group/condition, given as a discrete number and unit of measurement
☐	☒	A statement on whether measurements were taken from distinct samples or whether the same sample was measured repeatedly
☐	☒	The statistical test(s) used AND whether they are one- or two-sided<br>*Only common tests should be described solely by name; describe more complex techniques in the Methods section.*
☐	☒	A description of all covariates tested
☐	☒	A description of any assumptions or corrections, such as tests of normality and adjustment for multiple comparisons
☐	☒	A full description of the statistical parameters including central tendency (e.g. means) or other basic estimates (e.g. regression coefficient) AND variation (e.g. standard deviation) or associated estimates of uncertainty (e.g. confidence intervals)
☐	☒	For null hypothesis testing, the test statistic (e.g. *F*, *t*, *r*) with confidence intervals, effect sizes, degrees of freedom and *P* value noted<br>*Give P values as exact values whenever suitable.*
☒	☐	For Bayesian analysis, information on the choice of priors and Markov chain Monte Carlo settings
☒	☐	For hierarchical and complex designs, identification of the appropriate level for tests and full reporting of outcomes
☒	☐	Estimates of effect sizes (e.g. Cohen's *d*, Pearson's *r*), indicating how they were calculated

Our web collection on statistics for biologists contains articles on many of the points above.

Software and code

Policy information about availability of computer code

Data collection	Flow cytometry data was acquired using FACS Diva (8.0). NGS data was generated using Illumina sequencing platforms.
Data analysis	Flow cytomety data was analyzed using FlowJo10, Statistics were performed using GraphPad Prism version 7, NGS track figures were generated using the UCSC browser. ChIP-seq data was aligned to the mouse genome assembly version of July 2017 (NCBI37/mm9), using Bowtie (Langmead et al., 2009). Peaks were called using MACS 2.2.5 (Zhang et al., 2008) with default parameters. RNA-seq data was processed and analyzed using featureCounts 1.5.0, R version 3.3.3. and DESeq2 2.1.14.1. The analysis of Hi-C data was performed using HiCUP pipeline version 0.5.10, R 3.4.1 (https://www.r-project.org), Bowtie 2.2.9, SAMtools 1.4., Juicer tools 1.8.9, HOMER 4.10.3, the HiCCUPS algorithm from the Juicer tools, open2c distiller-nf pipeline [https://github.com/open2c/distiller-nf], Python 3.8.13, BEDTools package version 2.27.1. The open2c distiller-nf pipeline [https://github.com/open2c/distiller-nf] was used to process Micro-C datasets. These were further analysed using pairtools (https://github.com/open2c/pairtools), Cross-score algorithm (https://github.com/glab-vbc/cross-score), Peaks in Cross-score profiles were called using the ggpmisc package (Pedro J. Aphalo, 2021; https://github.com/aphalo/ggpmisc); Eigen value decomposition to calculate compartment signals was performed with the eigs-cis program from the cooltools package (https://zenodo.org/record/5214125#.YhjAXJYo_mE)

For manuscripts utilizing custom algorithms or software that are central to the research but not yet described in published literature, software must be made available to editors and reviewers. We strongly encourage code deposition in a community repository (e.g. GitHub). See the Nature Portfolio guidelines for submitting code & software for further information.

Data

Policy information about availability of data

All manuscripts must include a data availability statement. This statement should provide the following information, where applicable:

- Accession codes, unique identifiers, or web links for publicly available datasets
- A description of any restrictions on data availability
- For clinical datasets or third party data, please ensure that the statement adheres to our policy

The RNA-seq, ChIP-seq, VDJ-seq, Micro-C and Hi-C data reported in this study (Supplementary Data 3) will be available at the Gene Expression Omnibus (GEO) repository under the accession number GSE210289.

Human research participants

Policy information about studies involving human research participants and Sex and Gender in Research.

Reporting on sex and gender	N/A
Population characteristics	N/A
Recruitment	N/A
Ethics oversight	N/A

Note that full information on the approval of the study protocol must also be provided in the manuscript.

Field-specific reporting

Please select the one below that is the best fit for your research. If you are not sure, read the appropriate sections before making your selection.

☒ Life sciences ☐ Behavioural & social sciences ☐ Ecological, evolutionary & environmental sciences

For a reference copy of the document with all sections, see nature.com/documents/nr-reporting-summary-flat.pdf

Life sciences study design

All studies must disclose on these points even when the disclosure is negative.

Sample size	No statistical test was used to determine sample size. Sample sizes used in this study were based on those in previous studies in the same field (e.g. https://doi.org/10.1038/s41586-020-2454-y). For mouse phenotype analysis, the sample size was dependent on litter size and genotype abundance. Littermates were used as control and at least 3 independent litters were analyzed. For RNA-seq, VDJ-Seq and Hi-C, we followed the standard to perform at least 2 replica experiments.
Data exclusions	No data was excluded from analysis.
Replication	All experiments were repeated at least two times, with the exception of anti-H3K27ac ChIP-seq which has been performed once, as this track was only used for calculation of compartment signals and no further analysis was performed, and only reproducible results are described in the paper.
Randomization	Experiments were not randomized as randomization was not relevant to this study. Age matched mice of the same genetic background were used.
Blinding	Investigators were not blinded during data collection and analysis as investigators needed to verify that each experiment includes mutant strains and matched controls.

Reporting for specific materials, systems and methods

We require information from authors about some types of materials, experimental systems and methods used in many studies. Here, indicate whether each material, system or method listed is relevant to your study. If you are not sure if a list item applies to your research, read the appropriate section before selecting a response.

Materials & experimental systems

n/a	Involved in the study	
☐	☒	Antibodies
☒	☐	Eukaryotic cell lines
☒	☐	Palaeontology and archaeology
☐	☒	Animals and other organisms
☒	☐	Clinical data
☒	☐	Dual use research of concern

Methods

n/a	Involved in the study	
☐	☒	ChIP-seq
☐	☒	Flow cytometry
☒	☐	MRI-based neuroimaging

Antibodies

Antibodies used

The following monoclonal antibodies were used for flow cytometric analysis of the mouse bone marrow:
Specificity / Clone / Label / Company / Concentration / Dilution
CD19 / 1D3 / Brilliant Violet 421 / BD / 1:300 / 0.2 mg/ml
B220 / RA3-6B2 / Brilliant Violet 786 / BD / 1:200 / 0.2 mg/ml
IgM / II/41 / PerCPeFluor710 / Invitrogen by Thermo Fisher Scientific / 1:300 / 0.2 mg/ml
IgD / 11-26c / APC / Invitrogen by Thermo Fisher Scientific / 1:2000 / 0.2 mg/ml
CD25 / PC61 / PE / BD Pharmingen / 1:500 / 0.2 mg/ml
CD117 (c-Kit) / 2B8 PE-Cy7 / Invitrogen by Thermo Fisher Scientific / 1:1000 / 0.2 mg/ml
IgMa / MA-69 / FITC / BioLegend / 1:1000 / 0.5 mg/ml
IgMb / AF6-78 / PE / BioLegend / 1:1000 / 0.2 mg/ml

Western blot or immunoprecipitation experiments:
anti-Wapl (rabbit polyclonal Ab, A960; Peters laboratory), anti-TBP (mouse mAb clone 3TF1-3G3; Active Motif), anti-H3K27ac (rabbit polyclonal Ab, ab4729; Abcam)

Validation

Commercial antibodies that were validated by the vendor were used for experiments. No additional validation was performed.
The validation of the antibodies could be found from the manufacturers online:
https://www.bdbiosciences.com/en-at/products/reagents/flow-cytometry-reagents/research-reagents/single-color-antibodies-ruo/bv421-rat-anti-mouse-cd19.562701
https://www.bdbiosciences.com/en-at/products/reagents/flow-cytometry-reagents/research-reagents/single-color-antibodies-ruo/bv786-rat-anti-mouse-cd45r-b220.563894
https://www.thermofisher.com/antibody/product/IgM-Antibody-clone-II-41-Monoclonal/46-5790-82
https://www.thermofisher.com/antibody/product/IgD-Antibody-clone-11-26c-11-26-Monoclonal/17-5993-82
https://www.bdbiosciences.com/en-at/products/reagents/flow-cytometry-reagents/research-reagents/single-color-antibodies-ruo/pe-rat-anti-mouse-cd25.553866
https://www.thermofisher.com/antibody/product/CD117-c-Kit-Antibody-clone-2B8-Monoclonal/25-1171-82
https://www.biolegend.com/en-us/products/fitc-anti-mouse-igma-1755?GroupID=BLG2658
https://www.biolegend.com/en-us/products/pe-anti-mouse-igmb-1745
https://www.activemotif.com/catalog/details/61329/tbp-antibody-mab-clone-3tf1-3g3
https://www.abcam.com/products/primary-antibodies/histone-h3-acetyl-k27-antibody-chip-grade-ab4729.html

Animals and other research organisms

Policy information about studies involving animals; ARRIVE guidelines recommended for reporting animal research, and Sex and Gender in Research

Laboratory animals	Mus musculus, the following mice were maintained on the C57BL/6 background: Wapl(ΔP1,2/ΔP1,2) mice, Rag2(–/–) mice and Igh (B1-8hi/B1-8hi) mice, male and female, 4 weeks to 6 weeks
Wild animals	This study did not involve wild animals
Reporting on sex	Male and female animals were used in this study
Field-collected samples	This study did not involve field-collected samples
Ethics oversight	Valid project licenses were approved and controlled by the Austrian Veterinary Authorities (mentioned in method section)

Note that full information on the approval of the study protocol must also be provided in the manuscript.

ChIP-seq

Data deposition

☒ Confirm that both raw and final processed data have been deposited in a public database such as GEO.

☒ Confirm that you have deposited or provided access to graph files (e.g. BED files) for the called peaks.

Data access links<br>*May remain private before publication.*	ChIP-seq data will be available at the Gene Expression Omnibus (GEO) repository under the accession number GSE210289.
Files in database submission	ChIP-seq samples are listed in Supplementary Data 3.
Genome browser session<br>(e.g. UCSC)	no longer applicable

Methodology

Replicates	anti-H3K27ac ChIP-seq has been performed once
Sequencing depth	Paired End 50bp<br>Total reads: 94.058.012<br>Mapped reads: 82.437.596
Antibodies	anti-H3K27ac (rabbit polyclonal Ab, ab4729; Abcam)
Peak calling parameters	Peaks were called using the callpeak command of MACS version 2.2.5, with parameters -m 5 50 --fix-bimodal -q 0.05 --call-summits --keep-dup 1 --bdg --SPMR -g 2.304.947.926. Sample GSM2461724 (pro-B cell input) was used as control.
Data quality	Peaks were called using the callpeak command of MACS version 2.2.5, with parameters -m 5 50 --fix-bimodal -q 0.05 --call-summits --keep-dup 1 --bdg --SPMR -g 2.304.947.926. Sample GSM2461724 (pro-B cell input) was used as control. This resulted in 17.935 peaks with at least one summit with fold change > 5 (and FDR<0.05).
Software	Peaks were called using the callpeak command of MACS version 2.2.5. H3K27ac ChIP-seq data were used to calculate compartment signals using eigs-cis program from the cooltools package (https://zenodo.org/record/5214125#.YhjAXJYo_mE)

Flow Cytometry

Plots

Confirm that:

☒ The axis labels state the marker and fluorochrome used (e.g. CD4-FITC).

☒ The axis scales are clearly visible. Include numbers along axes only for bottom left plot of group (a 'group' is an analysis of identical markers).

☒ All plots are contour plots with outliers or pseudocolor plots.

☒ A numerical value for number of cells or percentage (with statistics) is provided.

Methodology

Sample preparation	Single cell suspensions of freshly isolated bone marrow were prepared and passed through a 70 um mesh.
Instrument	FACS LSR Fortessa 2
Software	Data collection: FACS Diva (8.0)<br>Data analysis: FlowJo
Cell population abundance	The abundance of the post-sort fraction was determined by re-analysis.
Gating strategy	The live gate (FSC/SSC) included all live cells. Erythrocytes were gated out.

☒ Tick this box to confirm that a figure exemplifying the gating strategy is provided in the Supplementary Information.
