# Supplementary material for: Igh and Igk loci use different folding principles for V gene recombination due to distinct chromosomal architectures of pro-B and pre-B cells
Source: Nat Commun. 2023 Apr 21;14:2316. doi: 10.1038/s41467-023-37994-9 (PMC10121685; doi:10.1038/s41467-023-37994-9)
Supplement: Supplementary file 4 — Description of Additional Supplementary Files [file 41467_2023_37994_MOESM4_ESM.docx]

**Description of additional supplementary files (Hill et al., 2023)**

**Supplementary Data 1**. List of all annotated V_K_ genes of the *Igk* locus (**a**) on mouse chromosome 6 and all V_H_ genes of the *Igh* locus (**b**) on mouse chromosome 12, based on the publication of Proudhon et al. (Adv. Immunol. 128, 123-182). The orientation, mm9 genomic coordinates and functional classification are shown for each V_K_ gene (**a**). The mm9 and mm10 genomic coordinates are shown for each V_H_ gene (**b**).

**Supplementary Data 2**. List of the 61 upregulated and 17 downregulated genes identified by RNA-seq analysis of *Wapl*^∆P1,2/∆P1,2^ and *Wapl*^+/+^ pre-B cells. Differentially regulated genes were defined by an expression difference of > 2-fold, an adjusted *P* value of < 0.05 and a TPM value of > 5 in at least one of the two pre-B cell types.

**Supplementary Data 3**. List of all VDJ, Hi-C and Micro-C sequencing experiments, which were performed for this study, and of previously published Illumina sequencing experiments. All sequencing data analyzed in this study are available at the Gene Expression Omnibus (GEO) repository under the accession number GSE201289.

**Supplementary Movie 1. Continuous remodeling of chromatin loops within the V_K_ gene region of the *Igk* locus in pre-B cells.** The presence of forward and reverse CTCF-binding elements (CBEs) along the V_K_ gene cluster results in the formation of multiple different loops and thus in the collision of their cohesin rings (orange) in response to ongoing loop extrusion. As a consequence, a transient interaction zone (orange) is formed that juxtaposes different DNA sequences (here shown as sequences 1-5 or 6-10) at the base of these loops next to the DNA sequences (gray) containing the two CBEs (black arrowhead) of the Cer element (Supplementary Fig. 1a), which facilitates their crosslinking and defines specific interactions along the stripe emanating from the Cer region in the Micro-C contact matrix of the *Igk* locus (Fig. 5d). Supplementary Video 1 shows how the loop pattern in the V_K_ gene cluster is constantly turned over so that new loops present different DNA sequences in the interaction zone, which results in a contiguous stripe consisting of all possible interactions along the V_K_ gene cluster in the large population of one million pre-B cells used for Micro-C analysis (Fig. 5a,b). Due to high Wapl expression in pre-B cells, continuous remodeling of the loops is likely caused by Wapl-mediated opening of cohesin rings, continuous loop extrusion of still existing cohesin rings, the initiation of new loops and ongoing loop extrusion, which generates a new interaction zone. Gray shading highlights the stable regulatory loop (Supplementary Fig. 7c) that contains the J_K_, C_K_ and *Igk* enhancer elements and is formed by the convergent alignment of the two forward-oriented CBEs (red arrowhead) of the Sis element and the next reverse-oriented downstream CBE (gray arrowhead; Supplementary Fig. 1a).
